# PPAR*γ* Inhibitors as Novel Tubulin-Targeting Agents

**DOI:** 10.1155/2008/785405

**Published:** 2008-05-26

**Authors:** Katherine L. Schaefer

**Affiliations:** Gastroenterology and Hepatology Division, Department of Medicine, University of Rochester Medical Center, Rochester, NY 14642, USA

## Abstract

The microtubule-targeting agents (MTAs) are a very successful class of cancer drugs with therapeutic benefits in both hematopoietic and solid tumors. However, resistance to these drugs is a significant problem. Current MTAs bind to microtubules, and/or to their constituent tubulin heterodimers, and affect microtubule polymerization and dynamics. The PPAR*γ* inhibitor T0070907 can reduce tubulin levels in colorectal cancer cell lines and suppress tumor growth in a murine xenograft model. T0070907 does not alter microtubule polymerization in vitro, and does not appear to work by triggering modulation of tubulin RNA levels subsequent to decreased polymerization. This observation suggests the possible development of antimicrotubule drugs that work by a novel mechanism, and implies the presence of cancer therapeutic targets that have not yet been exploited. This review summarizes what is known about PPAR*γ* inhibitors and cancer cell death, with emphasis on the tubulin phenotype and PPAR-dependence, and identifies potential mechanisms of action.

## 1. INTRODUCTION

The peroxisome proliferator-activated receptors (PPARs) are ligand-activated nuclear hormone receptors that act as transcriptional modulators. They have important roles in control of metabolism, inflammation, and cell growth and differentiation. There are three PPAR isoforms (*γ*, *β*/*δ*, and *α*) with overlapping but distinct tissue expression patterns and cellular functions [1]. Much evidence suggests that PPAR*γ* activity can modulate tumor development, implicating PPAR*γ* as an important therapeutic cancer target [2].

PPAR*γ* (NR1C3) is able to both activate and repress transcription, depending on the promoter that is involved [3]. In the classical pathway, PPAR*γ* binds to promoters containing PPAR-response elements (PPREs) in combination with its heterodimer partner, the retinoid X receptor. Activator ligand binding to PPAR*γ* causes a structural shift that increases its ability to recruit transcriptional coactivators while decreasing its basal ability to bind to corepressors [4]. PPAR*γ* also exhibits transrepressive functions at promoters lacking a PPRE [5], by binding in a ligand-dependent manner to transcription factors, cofactors, or repressor complexes. In these cases, PPAR*γ* binding inhibits transcription, either by binding/sequestering the transcription factors or by preventing clearance of repressor complexes. In at least one case of transrepression, the specific PPAR*γ* conformational shift required is different from that required for cofactor recruitment at PPREs [6]. It is worth noting here that since PPAR*γ* has basal ligand-independent repression [5] and activation functions [3], the effects of PPAR*γ* inhibitor binding and PPAR*γ* knockdown may not be the same.

PPAR*γ* can be activated pharmacologically by thiazolidenedione (TZD) compounds, including the antidiabetic drugs pioglitazone and rosiglitazone. There are multiple studies showing that high doses of TZDs can inhibit tumor growth in cell lines and mouse models. Clinical trials are currently underway testing TZDs as chemopreventive and therapeutic agents in human cancers [11]. While TZDs act to stimulate PPAR*γ* activity, they also have multiple PPAR*γ*-independent effects, and the specific role of PPAR*γ* activation itself in the therapeutic effects of TZDs is still an active area of research. These topics are reviewed, from the point of view of cancer therapeutic effects, in several recent reviews [11–18] and elsewhere in this special issue of *PPAR research.*

Several studies indicate that PPAR*γ* inhibitor compounds are also able to reduce tumor growth in preclinical models [9, 19–29]. As with the TZDs, the precise role of the loss of PPAR*γ* activity in cell death is an active research area, and may depend on the specific cell type. Our recent observation that PPAR*γ* inhibitors can cause rapid dissolution of the microtubule network in colon cancer cells [26] suggests that these compounds might act as microtubule-targeting agents (MTAs), similar to the taxanes or *Vinca* alkaloids that are in current clinical use. However, unlike MTAs [30], they markedly reduce concentrations of *α* and *β* tubulin proteins long before a commitment to apoptosis, and do not strongly affect microtubule polymerization in vitro. This review will focus on the strong possibility that PPAR*γ* inhibitor compounds represent a new class of tubulin-targeting agents [31].

## 2. BINDING ACTIVITY OF PPAR*γ* ACTIVATORS AND INHIBITORS

The PPAR*γ* ligand-binding pocket can accommodate a variety of lipophilic molecules [32]. Many cellular fatty acids activate PPAR*γ*, including oxidized low-density lipoproteins, unsaturated fatty acids, 15-hydroxyeicosatetraenoic acid, and 9- and 13-hydroxyoctadecadienoic acids. In addition, the putative endogenous ligand prostaglandin 15-deoxy delta-(12,14)-prostaglandin J2, as well as the TZD anti-diabetic drugs [32], are able to activate PPAR*γ*. The anti-inflammatory drug 5-aminosalicylic acid binds to PPAR*γ* at therapeutic doses [33], as do other nonsteroidal anti-inflammatory drugs [34], although both classes of medications are lower affinity ligands than the TZDs. Ligand binding introduces PPAR*γ* conformational shifts that favor recruitment of transcriptional coactivators over corepressors or that promote specific posttranslational modifications, and it is these changes that dictate the transcriptional activity of PPAR*γ*. All of these ligands also have multiple effects that are independent of PPAR*γ*, especially at high doses [13, 32]. In addition, the identity and regulation of true endogenous ligands is poorly understood at the present time.

PPAR*γ* also binds to a number of compounds that are able to inhibit TZD-mediated PPAR*γ* activation (see [35] for chemical structures). These include halofenate [36] and its enantiomer metaglidasen [37], SR-202 [38], G3335 and its derivatives [35, 39], T0070907 [9], GW9662 [8], and bisphenol-A-diglycidyl-ether (BADGE) [10]. PPAR*γ* inhibitors probably suppress PPAR*γ* activation both by preventing binding by endogenous or exogenously added ligands, and by inducing specific conformational shifts that actively promote repression [9]. However, the details of these conformational changes are less well understood than for the activators. Of the known PPAR*γ* inhibitors, only T0070907, GW9662, and BADGE have been tested for their effects on cancer cell death; all three can cause cell death in multiple cancer cell types at high-micromolar concentrations.

Interpreting the effects of the cancer-targeting PPAR*γ* inhibitors is difficult, since they can act as activators or inhibitors, depending on the concentration used. They also bind to multiple members of the PPAR family (and quite possibly to other molecules) at high doses. At low micromolar doses, T0070907 and GW9662 also bind to and inhibit PPAR*α* and PPAR*δ* (Table 1). In addition, at low nanomolar doses, GW9662 is a partial activator of PPAR*α*. While the ability of T0070907 to activate PPAR*α* has not been checked, it is possible that this compound may behave in the same manner. Similarly, there are reports that BADGE can act as a PPAR*γ* activator at lower doses (10–30 *μ*M) than those needed for inhibitory effects [28, 40].

## 3. STANDARD MICROTUBULE TARGETING AGENTS ACT BY INTERFERING WITH MICROTUBULE DYNAMICS

**Figure 1:**
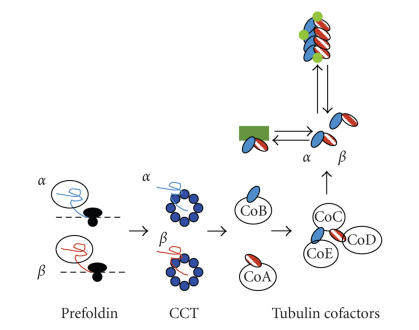
Microtubule formation depends both on chaperone-mediated production and assembly of *α*/*β* heterodimers and on microtubule-associated proteins. Production of *α* and *β* tubulin proteins requires assistance from chaperone proteins. The chaperone prefoldin associates with nascent tubulin polypeptide chains and delivers them to the CCT chaperone. CCT folds them into stable forms, which are delivered to the tubulin cofactors A and B [47]. CoA and CoB both transfer tubulin monomers to the CoC/D/E complex, which assembles the monomers into *α*/*β* heterodimers ready for introduction into microtubules. Tubulin reservoirs are held by the microtubule-associated protein stathmin (green box), which, depending on phosphorylation, binds to free tubulin and also destabilizes microtubule polymers. A host of micotubule-associated proteins (green circles) associate with the microtubule and regulate addition and removal of heterodimers from both ends of the microtubule; in some cases, they have been shown to regulate tubulin levels. While MTA therapies like the taxanes and *Vinca* alkaloids target the equilibrium between *α*/*β* tubulin heterodimer and the microtubule polymer, PPAR*γ* inhibitors could be affecting any of the chaperone proteins or one of the microtubule-associated proteins that is involved in control of tubulin levels.

Microtubules are long, tube-shaped polymers, formed by ordered arrays of *α*/*β* tubulin heterodimers (Figure 1), that make up one of the major components of the cellular cytoskeleton. Precise regulation of microtubule function is essential for maintenance of cell shape and polarity, migration, regulation of cell signaling cascades, intracellular transport, and cell division [41]. Microtubule function is governed by a variety of active changes in microtubule structure collectively termed *microtubule dynamics*. Microtubules normally alternate between phases of growth and rapid shrinking (dynamic instability), and also move tubulin heterodimers from one end of the polymer to the other (treadmilling) [42]. These processes are regulated in the cell by a host of microtubule-associated proteins with varied functions [43–46]. Both dynamic instability and treadmilling are required for mitosis, and are almost certainly necessary for the other functions of microtubules [44].

Given the importance of rapid cell proliferation and migration to tumor development, it is not surprising that the microtubule-targeting agents (MTAs) are one of the most successful classes of cancer therapeutics, with clinical applications in hematological cancers and solid tumors of the head/neck, breast, ovaries, testes, lung, gastric tissue, and prostate [48]. MTAs are a chemically and structurally diverse sets of small molecules that bind to microtubules, tubulin, or both, and interfere with microtubule function [44]. Despite this diversity, all known MTAs bind at or near to one of three domains: the taxane domain, the *Vinca* domain, or the colchicine domain. Historically, MTAs have been divided into microtubule-stabilizing and microtubule-destabilizing agents, based on their effects at high doses on polymer mass in in vitro polymerization assays. However, while the classification remains in use, and these effects clearly occur in vivo at high doses, it is becoming generally accepted that MTAs at clinically relevant concentrations primarily act by disrupting microtubule dynamics, rather than by affecting bulk polymerization [30, 49]. The microtubule-disrupting effect leads to cell cycle arrest. In addition, MTAs may also cause apoptosis by mechanisms that ultimately prove to be at least partially independent of the effect on the mitotic spindle [50, 51].

Discovery and development of new microtubule-binding compounds is an area of active research. In contrast, less effort has been spent on considering whether reducing tubulin levels directly, and thereby altering microtubule function, could be used to impair cancer cells. Our recent results, showing that PPAR*γ* inhibitors reduce tubulin levels in HT-29 colon cancer cells, before a commitment to apoptosis [26], suggest that targeting tubulin itself may be a viable strategy.

## 4. PPAR*γ* INHIBITORS CAUSE APOPTOSIS IN MULTIPLE CANCER CELL TYPES AND CAUSE RAPID LOSS OF TUBULIN PROTEINS IN COLON CANCER CELLS

Experiments with many different cancer cell lines show that high doses of PPAR*γ* inhibitors can cause cell death. T0070907 and/or GW9662 exhibited antiproliferative effects in both hematopoietic cell lines from non-Hodgkin's lymphoma and multiple myeloma [27] and epithelial cancer cell lines, including carcinoma cell lines from renal [27], breast [21, 22, 27], liver [23, 52], oral squamous [29], esophageal [24], prostate [22], and colon tissue [22, 26]. IC50 concentrations for inhibition of growth in the epithelial lines ranged from 3–50 *μ*M for T0070907 and 10–50 *μ*M for GW9662. While the reasons for this reduction in cell number have not been explored in all cases, T0070907 and GW9662 clearly caused apoptosis in several epithelial cell lines. BADGE also exhibited cytotoxic effects against colon cancer cell lines [20, 22, 25, 31] and a T lymphoma cell line [19, 20], at doses in the 100–200 *μ*M range.

In HT-29 colon cancer cells, treatment with 50 *μ*M T0070907 led to dissolution of the microtubule network within 12 hours [26]. At this timepoint, the effects of T0070907 were reversible. However, after longer exposure, the cells became committed to caspase-dependent apoptosis. Cells treated with T0070907 also assumed a rounded shape that occurred prior to commitment to apoptosis, and that was not affected by caspase inhibitors. Similar effects and timing were seen with the PPAR*γ* inhibitor GW9662 ([26] and KLS, unpublished data).

Loss of the microtubule network is associated with the microtubule-depolymerizing class of MTAs that includes vinblastine, vincristine, vinorelbine, and nocodazole [30, 53]. Thus, it was especially striking that, unlike nocodazole, T0070907 and GW9662 did not affect microtubule polymerization in in vitro assays [26]. Instead, *α* and *β* tubulin protein levels in the cells dropped rapidly after treatment with these compounds, suggesting that the microtubule network disappeared because tubulin protein levels were below critical thresholds needed for polymerization. BADGE also caused tubulin loss, although the timing of this loss relative to commitment to apoptosis was not determined. It is not currently known whether PPAR*γ* inhibitors cause loss of tubulin in other cell lines. While many of the other experiments with inhibitors documented altered cytoskeletal structure [23–25, 29], it was not clear in these papers whether the altered shape was the result or the cause of apoptosis, and tubulin levels were not measured directly.

## 5. IS TUBULIN LOSS REQUIRED FOR CELL DEATH INDUCED BY T0070907 AND GW9662?

The effects of T0070907 and GW9662 on tubulin are striking, especially as there have not been any reports of cancer cell-targeting small molecules that affect tubulin levels without dramatic effects on microtubule polymerization. However, it is not clear that the ultimate cause of cell death is loss of tubulin. These compounds may independently target a combination of signaling pathways that ultimately trigger the apoptotic response as well as modulating tubulin levels. Given the fact that the PPAR*γ* inhibitors also trigger loss of *γ* and *δ* tubulin isoforms (KLS, unpublished data), it will be difficult to do genetic replacement experiments to address this issue in the absence of other information about the reasons for tubulin loss. Regardless of whether or not microtubule disruption is the first trigger for apoptosis, the reduction in tubulin should serve as a barrier that is impossible for the tumor cells to surmount. This effect could have profound advantages, in that simply modulating the levels of anti-apoptotic proteins in response to chemotherapy would not be sufficient to allow tumor escape.

Intriguingly, commitment to apoptosis in HT-29 cells occurred at about the same time that tubulin levels dropped below a threshold level for normal function observed in yeast. After 12 hours of T0070907 treatment, when the tumor cells had lost ∼50% of their tubulin, the effects of the drug were still reversible. By the time, 50% of the cells had committed to apoptosis (∼20 h), the average tubulin level was less than 10% of control ([26] and data not shown), suggesting that apoptosis may be triggered by tubulin levels below 50% of normal. These numbers parallel observations in the yeast *Saccharomyes cerevisiae*, which was able to tolerate a 50% reduction in either *α* or *β* tubulin, as long as there were not excess unpaired *β* tubulin molecules [54, 55], but which began to show defects in mitosis when levels dropped to ∼20% of normal.

## 6. WHAT COULD BE CAUSING THE LOSS OF TUBULIN INDUCED BY T0070907?

The reasons for T0070907-mediated tubulin loss remain to be elucidated, and may well be the result of multiple coordinated changes taking place in the context of alterations in PPAR function. This point is of critical interest, as identification of the mechanism(s) of tubulin loss will serve as an important step in identifying the therapeutic targets that are exploited by T0070907, and in design of better ways to target them. In HT-29 cells, *α*/*β* tubulin RNA levels were unaffected, suggesting a post-transcriptional mechanism. Several aspects of tubulin production could be involved, including degradation, translation initiation, chaperone-mediated folding/assembly of tubulin heterodimers (Figure 1), and disruption of microtubule-associated protein interactions with tubulin.

Because the protein half-life of tubulin is believed to be long (∼50 hours) [56, 57], detectable loss of tubulin within 6 hours suggests a mechanism involving increased decay. Tubulin can be targeted for proteasomal degradation by the tubulin cofactor-like protein El [58] and probably by other regulatory factors. However, proteasome inhibitors did not prevent T0070907-induced tubulin loss [26], suggesting that ubiquitin-mediated proteasomal degradation is not a major factor. Other degradation pathways must be investigated, especially the aggresome pathway, which can replace proteasomal degradation, resulting in autophagic clearance and lysosomal degradation [59, 60]. It is also worth noting that the estimate of long tubulin half-lives is based on measurement in only two cell types, and may not apply to HT-29 cells.

T0070907-induced tubulin loss is unlikely to be the result of acute increases in tubulin monomer protein. Many eukaryotic cells respond to a sudden increase in unpolymerized tubulin by reducing synthesis of tubulin [61–65] in a process termed *autoregulatory control*. This mechanism is associated with large reductions in tubulin mRNA; later work showed that polysomal mRNA in the process of being translated was specifically susceptible to an increased mRNA decay [66, 67]. T0070907 induces little difference in tubulin mRNA concentrations as measured by real-time PCR [26]. In addition, at least in in vitro polymerization assays, T0070907 did not inhibit polymerization or cause depolymerization, although it is important to note that depolymerization might occur in the cell as a result of alterations in microtubule-associated protein function. However, nocodazole, which at high (10 *μ*M) doses increases the amount of soluble tubulin by inhibiting bulk microtubule polymerization [53], did not affect the tubulin protein levels in HT-29 cells. This result strongly suggests that tubulin in these particular cells is not strongly subject to autoregulatory control. However, it is formally possible that T0070907 might increase the soluble tubulin pool far more strongly than nocodazole. Direct measurement of the amount of tubulin in the polymerized and free pools after T0070907 treatment should resolve these questions.

There is also the potential for T0070907 to control translation initiation or other aspects of protein synthesis. The TZD PPAR*γ* activators have been shown to suppress translation initiation in a PPAR*γ*-independent manner through a mechanism involving intracellular Ca^2+^ store depletion and subsequent inhibition of the eIF2 translation inititation factor [68]. It is possible that T0070907 also affects some aspect(s) of the translation machinery.

The loss of tubulin and cell death phenotypes induced by T0070907 can be mimicked by knockdown of chaperone proteins involved in folding and assembly of tubulin, suggesting that PPAR*γ* inhibitors may interfere with this pathway. Tubulin production and assembly into *α*/*β* heterodimers require the presence of multiple chaperone proteins, including the multisubunit chaperones prefoldin [69] and CCT [70], and tubulin cofactor proteins A–E [71] (Figure 1). Additional chaperone modulatory proteins, including PhLP3 [72] and E-like (El) [58], also modulate the function of the tubulin chaperone system. Knockdown of CCT subunits causes reduced tubulin levels [73], as does knockdown of tubulin cofactor A [74]. It is possible that PPAR*γ* inhibitors bind directly to and inhibit some of the chaperone proteins. Alternatively, they may change the expression or function of any of the chaperone subunits or cofactors.

Knockdown of microtubule-associated proteins (MAPs) can also cause loss of tubulin, and PPAR*γ* inhibitors could interfere with MAPs or dysregulate MAP expression. MAPs are a functional class of proteins that physically interact with microtubules or microtubule precursors and regulate microtubule functions. Some MAPs directly control the rate of association or dissociation of *α*/*β* tubulin heterodimers with the ends of the microtubule, as well as the levels of soluble tubulin in the cell, and thus affect microtubule dynamics (Figure 1). Others link microtubules to signaling complexes and other cytoskeletal components [75]. Mutations in stathmin, a multifunctional MAP that both destabilizes microtubules and sequesters *α*/*β* tubulin heterodimers so that they are not part of the freely polymerizing pool, led to reduced *α* tubulin levels (*β* tubulin was not checked) and fewer microtubules in Drosophila oocytes [76]. In the same system, stathmin overexpression increased tubulin levels. Knockdown of MAP4, generally thought to be a microtubule-stabilizing MAP, also caused reduced tubulin levels [77]. In both cases, it is possible, but has not been shown directly, that these effects were subsequent to autoregulatory control.

## 7. ARE THE CELL DEATH AND TUBULIN EFFECTS OF T0070907 OR GW9662 DEPENDENT UPON PPAR*γ*?

It is important to establish whether the effects of T0070907 and GW9662 on cell death can be separated from their ability to target PPAR*γ*. Although results in two cell lines [23, 29] have shown that PPAR*γ* knockdown causes cell death or potentiates the effects of the inhibitors, this result does not occur in all cell lines [26]. In addition, the inhibitor doses required for the cell death (3–50 *μ*M for T0070907 and 10–50 *μ*M for GW9662) in all cell lines tested are much higher than those needed to inhibit the transcriptional effect of PPAR*γ* by at least 90%, given an approximate effective dissociation constant in the low nanomolar range (Table 1). This result suggests that at least some of the cell death effects are indeed independent of PPAR*γ*. The differences in cell lines may reflect a true disparity in the role of PPAR*γ* in maintaining cell growth and survival in different cell types, and suggests that the HT-29 system is ideal for examining the PPAR*γ*-independent effects of T0070907.

The effect of T0070907 and GW9662 on tubulin has been examined primarily in HT-29 colon cancer cells, although the loss of adhesion, cell rounding, and cell death occur in multiple colorectal cancer cell lines ([26] and KLS, unpublished data). In HT-29 cells, the effects on tubulin are not replicated by PPAR*γ* knockdown, or reductions in the closely related PPAR*δ*. However, given the fact that at 50 *μ*M T0070907, PPAR*γ*, PPAR*α* and PPAR*δ* transcriptional activities are all expected to be at least partially repressed, based on the predicted dissociation constants (Table 1), it is possible that multiple PPAR molecules must be inactivated in order to create the conditions necessary for tubulin loss. In addition, it is entirely possible that a non-PPAR-dependent event must occur in the context of knockdown of one or more of the PPAR transcription factors. This idea is somewhat contradicted by the observation that BADGE, which does not strongly affect PPAR*α*/*δ* at 100 *μ*M [10], does cause a reduction in the amount of tubulin [26]. However, in this case, it is possible that the tubulin loss was a separate phenomenon, secondary to extensive cell death. Further experiments will be needed to address this issue.

## 8. ADVANTAGES AND DISADVANTAGES TO USING PPAR*γ* INHIBITORS AS TUBULIN-TARGETING THERAPIES

To our knowledge, the PPAR*γ* inhibitors are the first described instance of a possible small molecule cancer therapeutic that reduces tubulin levels. This result suggests the exciting possibility that tubulin levels can be modulated therapeutically, in a tumor-specific manner, to kill cancer cells. While the current microtubule-targeting agents have significant antitumor activity in many cancer types, they are not effective in all cancers, and acquired resistance is a problem [78]. In addition, because these drugs differentially targeting rapidly proliferating cells, they cause leukopenia [78]. For the same reason, they are not expected to target cancer stem cells, owing to their generally slow proliferation rate [79]. Microtubule-targeting drugs also induce peripheral neuropathy [80] and may interfere with mental function [81], presumably as a result of their effects on neuronal microtubule function. T0070907 and/or GW9662, or second-generation compounds, by virtue of acting by a different mechanism, might ameliorate some of the difficulties of the standard MTAs.

Several questions must be addressed, when considering these compounds, or others like them, as cancer therapies. To date, preclinical data on the bioavailability of either T0070907 or GW9662 has not been published, although a pilot experiment with radiolabeled GW9662 indicates that these compounds are delivered to tumors [82]. It is also important to consider tumor specificity and whether the tubulin-targeting effect can be separated from the PPAR inhibitory effect.

Inherent or acquired resistance to current microtubule-targeting agents is a serious problem in microtubule-based cancer therapy. One major source of resistance is the expression of alternate tubulin isoforms with inherently different microtubule dynamics [83]. These differences antagonize the effects of the drug, and allow the cell to continue proliferating. It appears that T0070907 causes concurrent loss of multiple tubulin isoforms (KLS, unpublished data), presumably by some regulatory mechanism common to all isoforms. It is therefore reasonable to suspect that T0070907 would suppress levels of the tumor-specific alternate isoforms as well. The microtubule targeting drugs are also generally good substrates for drug efflux pumps that prevent accumulation of therapeutic levels of drug [78]. For this reason, it would be useful to test whether T0070907 and GW9662 are substrates for the common drug efflux pumps.

As tubulin is a constituent of all cells, the effects of T0070907 or similar compounds on normal cells is a serious consideration. To date, these compounds have only been tested in one mouse model of cancer. At oral doses that reduced tumor growth (1–5 mg/kg/d), the compounds did not cause weight loss or malaise in mice [26]. In addition, recent unpublished results from our laboratory showed that 10 mg/kg/d orally, maintained daily for three weeks, did not cause any alterations in values from a standard complete blood count with differential. The reasons for this apparent specificity will need to be examined in more detail, as well as whether tubulin levels are reduced in normal and tumor tissues in vivo. The fact that radioactively labeled GW9662 preferentially accumulated in tumor cells as compared to many normal tissues in mice [82] suggests that part of the specificity may simply reflect where the compound is accumulating. Another possibility is that these compounds do act through the tubulin chaperone system. Components of this system are upregulated in some tumor cells [84–86], and tumor cells may require higher levels of tubulin chaperone function, as they do with the HSP90 chaperone [87, 88]. If this were true, this might explain why tumor cells are preferentially susceptible to the PPAR*γ* inhibitors.

A major question is whether suppression of PPAR*γ* (or PPAR*α*/*δ*) function is required for the tubulin-targeting effects of T0070907 and/or GW9662. If suppression of PPAR*γ* cannot be separated from the tubulin targeting effects, it will be necessary to carefully balance the therapeutic effects of PPAR*γ* inhibitors on tubulin with the possible deleterious effects of PPAR*γ* inhibition on physiologic processes that affect tumor growth. In addition to the question of whether PPAR*γ* is a tumor suppressor or has tumor-promoting activity in each cancer cell type, the effects of PPAR*γ* on angiogenesis [14, 16] and the immune system [32] must also be considered.

The role of PPAR*γ* itself in angiogenesis, in contrast to the effects of TZD PPAR*γ* activators, is still relatively unclear [16]. TZDs can reduce the production of proangiogenic FGF and VEGF factors, interfere with endothelial cell migration, and inhibit vascular tube formation, as well as reduce production of inflammatory mediators that stimulate angiogenesis. However, to date, only CD36 upregulation [89] and decreased iNOS production [6] are known to be PPAR*γ*-dependent. It is also noteworthy that standard microtubule-targeting agents in clinical use disrupt tumor-specific vasculature [90, 91]. It will be interesting to determine whether the PPAR*γ* inhibitors also suppress tumor angiogenesis, and whether the effects are linked to PPAR*γ* and/or tubulin.

In all probability, the net effect of PPAR*γ* inhibition on the immune system will depend upon the individual characteristics of the tumor, including the site of the tumor, the role of PPAR*γ* in tumor-intrinsic biology, and the presence and type of immune infiltration. A variety of immune cells infiltrate tumors, including macrophage lineage cells, T lymphocytes, mast cells, and natural killer (NK) cells [92]. All of these cells have the potential to promote or hinder tumor growth, depending on the cytokines they secrete and the cell-mediated cytotoxic effects they are able to promote. Both macrophage and regulatory T cell (T_reg_) functions are modulated by PPAR*γ*. Monocytes can differentiate into M1 or M2 macrophages in response to different stimuli [93, 94]. In general, M1 macrophages secrete large quantities of inflammatory cytokines, have cytotoxic activity toward tumor cells, elicit the adaptive immune response, and are associated with a better tumor prognosis. In contrast, M2 macrophages secrete immunosuppressive cytokines, have poor antigen-presenting capacity, and promote angiogenesis and tissue remodeling; these macrophages are generally associated with a poorer prognosis [93]. Since PPAR*γ* activation of human monocytes promotes M2 polarization [95], PPAR*γ* inhibitors might be expected to favor production of M1 tumor-suppressing inflammatory macrophages. T_reg_ cells lacking PPAR*γ* are unable to suppress colitis in a regulatory T cell-dependent model of inflammatory bowel disease [96], arguing that PPAR*γ* is required for suppressive regulatory function. As T_reg_ activity can impair tumor rejection [97], PPAR*γ* inhibitors should suppress T_reg_ thereby aiding tumor rejection.

## 9. CONCLUSIONS

The microtubule-targeting agents are one of the most successful classes of cancer therapeutics, but ongoing issues with resistance make the development of additional strategies for targeting microtubules extremely desirable. The recent discovery that the small molecule PPAR*γ* inhibitor compounds reduce tubulin protein levels, without affecting in vitro polymerization rates, suggests the exciting possibility that targeting tubulin levels directly, rather than microtubule dynamics, might be an additional way to manipulate microtubule biology to kill cancer cells. Several questions, including whether inhibition of PPAR function is required for the tubulin effect, the nature of the tumor specificity, the ultimate targets of these compounds, and whether better compounds with similar tubulin targeting effects can be designed, must be answered before this strategy can be fully realized.

## Figures and Tables

**Table 1:** Effects of PPAR*γ* inhibitors on PPAR*γ*, PPAR*α*, and PPAR*δ* activity IC50 (nM) for ability to compete with a PPAR agonist.

	Binding	Direct binding assay			Activation of GAL4 chimera			References.
		PPAR*γ*	PPAR*α*	PPAR*δ*	PPAR*γ*	PPAR*α*	PPAR*δ*	
GW9662	Irreversible				4	188	471	[7]
		5	39	1200	8	630^(1)^	4100	[8]
T0070907	Irreversible	1	850	1800	1 *μ*M completely inhibits *γ* with no effect on *α* or *δ* ^(2)^			[9]
BADGE	Reversible	100 000			100 *μ*M ∼70% inhibits *γ* with little or no effect on *α* or *δ* ^(2)^			[10]

^(1)^GW9662 is also a partial activator of PPAR*α* with an EC50 of 22 nM [8], leading to the apparently higher concentrations of GW9662 required to inhibit PPAR*α* than would be predicted by the direct binding assay. ^(2)^Dose curves were not performed, but the indicated concentrations suppressed the GAL4 chimera as indicated.

## References

[1] Tan NS, Michalik L, Desvergne B, Wahli W (2005). Multiple expression control mechanisms of peroxisome proliferator-activated receptors and their target genes. *Journal of Steroid Biochemistry and Molecular Biology*.

[2] Han S, Roman J (2007). Peroxisome proliferator-activated receptor *γ*: a novel target for cancer therapeutics?. *Anti-Cancer Drugs*.

[3] Feige JN, Gelman L, Michalik L, Desvergne B, Wahli W (2006). From molecular action to physiological outputs: peroxisome proliferator-activated receptors are nuclear receptors at the crossroads of key cellular functions. *Progress in Lipid Research*.

[4] Zoete V, Grosdidier A, Michielin O (2007). Peroxisome proliferator-activated receptor structures: ligand specificity, molecular switch and interactions with regulators. *Biochimica et Biophysica Acta*.

[5] Pascual G, Glass CK (2006). Nuclear receptors versus inflammation: mechanisms of transrepression. *Trends in Endocrinology & Metabolism*.

[6] Pascual G, Fong AL, Ogawa S (2005). A SUMOylation-dependent pathway mediates transrepression of inflammatory response genes by PPAR-*γ*. *Nature*.

[7] Seimandi M, Lemaire G, Pillon A (2005). Differential responses of PPAR*α*, PPAR*δ*, and PPAR*γ* reporter cell lines to selective 
PPAR synthetic ligands. *Analytical Biochemistry*.

[8] Leesnitzer LM, Parks DJ, Bledsoe RK (2002). Functional consequences of cysteine modification in the ligand binding sites of peroxisome proliferator activated receptors by GW9662. *Biochemistry*.

[9] Lee G, Elwood F, McNally J (2002). T0070907, a selective ligand for peroxisome proliferator-activated receptor *γ*, functions as an antagonist of biochemical and cellular activities. *Journal of Biological Chemistry*.

[10] Wright HM, Clish CB, Mikami T (2000). A synthetic antagonist for the peroxisome proliferator-activated receptor *γ* inhibits adipocyte differentiation. *Journal of Biological Chemistry*.

[11] Galli A, Mello T, Ceni E, Surrenti E, Surrenti C (2006). The potential of antidiabetic thiazolidinediones for anticancer therapy. *Expert Opinion on Investigational Drugs*.

[12] Chou F-S, Wang P-S, Kulp S, Pinzone JJ (2007). Effects of thiazolidinediones on differentiation, proliferation, and apoptosis. *Molecular Cancer Research*.

[13] Feinstein DL, Spagnolo A, Akar C (2005). Receptor-independent actions of PPAR thiazolidinedione agonists: is mitochondrial function the key?. *Biochemical Pharmacology*.

[14] Giaginis C, Margeli A, Theocharis S (2007). Peroxisome proliferator-activated receptor-*γ* ligands as investigational modulators of angiogenesis. *Expert Opinion on Investigational Drugs*.

[15] Koeffler HP (2003). Peroxisome proliferator-activated receptor *γ* and cancers. *Clinical Cancer Research*.

[16] Panigrahy D, Huang S, Kieran MW, Kaipainen A (2005). PPAR*γ* as a therapeutic target for tumor angiogenesis and metastasis. *Cancer Biology & Therapy*.

[17] Russu WA (2007). Thiazolidinedione anti-cancer activity: is inhibition of microtubule assembly implicated?. *Medical Hypotheses*.

[18] Weng J-R, Chen C-Y, Pinzone JJ, Ringel MD, Chen C-S (2006). Beyond peroxisome proliferator-activated receptor *γ* signaling: the multi-facets of the antitumor effect of thiazolidinediones. *Endocrine-Related Cancer*.

[19] Fehlberg S, Gregel CM, Göke A, Göke R (2003). Bisphenol A diglycidyl ether-induced apoptosis involves Bax/Bid-dependent mitochondrial release of apoptosis-inducing factor (AIF), cytochrome *c* and Smac/DIABLO. *British Journal of Pharmacology*.

[20] Fehlberg S, Trautwein S, Göke A, Göke R (2002). Bisphenol A diglycidyl ether induces apoptosis in tumour cells independently of peroxisome proliferator-activated receptor-*γ*, in caspase-dependent and -independent manners. *Biochemical Journal*.

[21] Seargent JM, Yates EA, Gill JH (2004). GW9662, a potent antagonist of PPAR*γ*, inhibits growth of breast tumour cells and promotes the anticancer effects of the PPAR*γ* agonist rosiglitazone, independently of PPAR*γ* activation. *British Journal of Pharmacology*.

[22] Lea MA, Sura M, Desbordes C (2004). Inhibition of cell proliferation by potential peroxisome proliferator-activated receptor (PPAR) gamma agonists and antagonists. *Anticancer Research*.

[23] Schaefer KL, Wada K, Takahashi H (2005). Peroxisome proliferator-activated receptor *γ* inhibition prevents adhesion to the extracellular matrix and induces anoikis in hepatocellular carcinoma cells. *Cancer Research*.

[24] Takahashi H, Fujita K, Fujisawa T (2006). Inhibition of peroxisome proliferator-activated receptor gamma activity in esophageal carcinoma cells results in a drastic decrease of invasive properties. *Cancer Science*.

[25] Ramilo G, Valverde I, Lago J, Vieites JM, Cabado AG (2006). Cytotoxic effects of BADGE (bisphenol A diglycidyl ether) and BFDGE (bisphenol F diglycidyl ether) on Caco-2 cells in vitro. *Archives of Toxicology*.

[26] Schaefer KL, Takahashi H, Morales VM (2007). PPAR*γ* inhibitors reduce tubulin protein levels by a PPAR*γ*, PPAR*δ* and proteasome-independent mechanism, resulting in cell cycle arrest, apoptosis and reduced metastasis of colorectal carcinoma cells. *International Journal of Cancer*.

[27] Burton JD, Castillo ME, Goldenberg DM, Blumenthal RD (2007). Peroxisome proliferator-activated receptor-*γ* antagonists exhibit potent antiproliferative effects versus many hematopoietic and epithelial cancer cell lines. *Anti-Cancer Drugs*.

[28] Bishop-Bailey D, Hla T, Warner TD (2000). Bisphenol A diglycidyl ether (BADGE) is a PPAR*γ* agonist in an ECV304 cell line. *British Journal of Pharmacology*.

[29] Masuda T, Wada K, Nakajima A (2005). Critical role of peroxisome proliferator-activated receptor *γ* on anoikis and invasion of squamous cell carcinoma. *Clinical Cancer Research*.

[30] Jordan MA (2002). Mechanism of action of antitumor drugs that interact with microtubules and tubulin. *Current Medicinal Chemistry: Anti-Cancer Agents*.

[31] Schaefer KL (2007). PPAR-*γ* inhibitors as novel tubulin-targeting agents. *Expert Opinion on Investigational Drugs*.

[32] Straus DS, Glass CK (2007). Anti-inflammatory actions of PPAR ligands: new insights on cellular and molecular mechanisms. *Trends in Immunology*.

[33] Rousseaux C, Lefebvre B, Dubuquoy L (2005). Intestinal antiinflammatory effect of 5-aminosalicylic acid is dependent on peroxisome proliferator-activated receptor-*γ*. *Journal of Experimental Medicine*.

[34] Lehmann JM, Lenhard JM, Oliver BB, Ringold GM, Kliewer SA (1997). Peroxisome proliferator-activated receptors *α* and *γ* are activated by indomethacin and other non-steroidal anti-inflammatory drugs. *Journal of Biological Chemistry*.

[35] Deng G, Liu Z, Ye F Tryptophan-containing dipeptide derivatives as potent PPAR*γ* antagonists: design, synthesis, biological evaluation, and molecular modeling.

[36] Allen T, Zhang F, Moodie SA (2006). Halofenate is a selective peroxisome proliferator-activated receptor *γ* modulator with antidiabetic activity. *Diabetes*.

[37] Zhang F, Lavan BE, Gregoire FM (2007). Selective modulators of PPAR-*γ* activity: molecular aspects related to obesity and side-effects. *PPAR Research*.

[38] Rieusset J, Touri F, Michalik L (2002). A new selective peroxisome proliferator-activated receptor *γ* antagonist with antiobesity and antidiabetic activity. *Molecular Endocrinology*.

[39] Ye F, Zhang Z-S, Luo H-B (2006). The dipeptide H-Trp-Glu-OH shows highly antagonistic activity against PPAR*γ*: bioassay with molecular modeling simulation. *ChemBioChem*.

[40] Nakamuta M, Enjoji M, Uchimura K (2002). Bisphenol A diglycidyl ether (BADGE) suppresses tumor necrosis factor-*α* production as a PPAR*γ* agonist in the murine macrophage-like cell line, RAW 264.7. *Cell Biology International*.

[41] Peterson JR, Mitchison TJ (2002). Small molecules, big impact: a history of chemical inhibitors and the cytoskeleton. *Chemistry & Biology*.

[42] Wilson L, Jordan MA (1995). Microtubule dynamics: taking aim at a moving target. *Chemistry & Biology*.

[43] Howard J, Hyman AA (2007). Microtubule polymerases and depolymerases. *Current Opinion in Cell Biology*.

[44] Jordan MA, Wilson L (2004). Microtubules as a target for anticancer drugs. *Nature Reviews Cancer*.

[45] Maccioni RB, Cambiazo V (1995). Role of microtubule-associated proteins in the control of microtubule assembly. *Physiological Reviews*.

[46] Morrison EE (2007). Action and interactions at microtubule ends. *Cellular and Molecular Life Sciences*.

[47] Grynberg M, Jaroszewski L, Godzik A (2003). Domain analysis of the tubulin cofactor system: a model for tubulin folding and dimerization. *BMC Bioinformatics*.

[48] Zelnak AB (2007). Clinical pharmacology and use of microtubule-targeting agents in cancer therapy. *Methods in Molecular Medicine*.

[49] Jordan MA, Kamath K (2007). How do microtubule-targeted drugs work? An overview. *Current Cancer Drug Targets*.

[50] Bhalla KN (2003). Microtubule-targeted anticancer agents and apoptosis. *Oncogene*.

[51] Estève M-A, Carré M, Braguer D (2007). Microtubules in apoptosis induction: are they necessary?. *Current Cancer Drug Targets*.

[52] Kim KR, Choi HN, Lee HJ (2007). A peroxisome proliferator-activated receptor *γ* antagonist induces vimentin cleavage and inhibits invasion in high-grade hepatocellular carcinoma. *Oncology Reports*.

[53] Vasquez RJ, Howell B, Yvon AM, Wadsworth P, Cassimeris L (1997). Nanomolar concentrations of nocodazole alter microtubule dynamic instability in vivo and in vitro. *Molecular Biology of the Cell*.

[54] Katz W, Weinstein B, Solomon F (1990). Regulation of tubulin levels and microtubule assembly in *Saccharomyces cerevisiae*: consequences of altered tubulin gene copy number. *Molecular and Cellular Biology*.

[55] Lacefield S, Magendantz M, Solomon F (2006). Consequences of defective tubulin folding on heterodimer levels, mitosis and spindle morphology in 
*Saccharomyces cerevisiae*. *Genetics*.

[56] Caron JM, Jones AL, Kirschner MW (1985). Autoregulation of tubulin synthesis in hepatocytes and fibroblasts. *Journal of Cell Biology*.

[57] Mooney DJ, Hansen LK, Langer R, Vacanti JP, Ingber DE (1994). Extracellular matrix controls tubulin monomer levels in hepatocytes by regulating protein turnover. *Molecular Biology of the Cell*.

[58] Bartolini F, Tian G, Piehl M, Cassimeris L, Lewis SA, Cowan NJ (2005). Identification of a novel tubulin-destabilizing protein related to the chaperone cofactor E. *Journal of Cell Science*.

[59] Hideshima T, Bradner JE, Wong J (2005). Small-molecule inhibition of proteasome and aggresome function induces synergistic antitumor activity in multiple myeloma. *Proceedings of the National Academy of Sciences of the United States of America*.

[60] Kopito RR (2000). Aggresomes, inclusion bodies and protein aggregation. *Trends in Cell Biology*.

[61] Ben-Ze'ev A, Farmer SR, Penman S (1979). Mechanisms of regulating tubulin synthesis in cultured mammalian cells. *Cell*.

[62] Caron JM, Jones AL, Rall LB, Kirschner MW (1985). Autoregulation of tubulin synthesis in enucleated cells. *Nature*.

[63] Cleveland DW, Havercroft JC (1983). Is apparent autoregulatory control of tubulin synthesis nontranscriptionally regulated?. *Journal of Cell Biology*.

[64] Cleveland DW, Lopata MA, Sherline P, Kirschner MW (1981). Unpolymerized tubulin modulates the level of tubulin mRNAs. *Cell*.

[65] Pittenger MF, Cleveland DW (1985). Retention of autoregulatory control of tubulin synthesis in cytoplasts: demonstration of a cytoplasmic mechanism that regulates the level of tubulin expression. *Journal of Cell Biology*.

[66] Gay DA, Sisodia SS, Cleveland DW (1989). Autoregulatory control of *β*-tubulin mRNA stability is linked to translation elongation. *Proceedings of the National Academy of Sciences of the United States of America*.

[67] Yen TJ, Machlin PS, Cleveland DW (1988). Autoregulated instability of *β*-tubulin mRNAs by recognition of the nascent amino terminus of 
*β*-tubulin. *Nature*.

[68] Palakurthi SS, Aktas H, Grubissich LM, Mortensen RM, Halperin JA (2001). Anticancer effects of thiazolidinediones are independent of peroxisome proliferator-activated receptor *γ* and mediated by inhibition of translation initiation. *Cancer Research*.

[69] Vainberg IE, Lewis SA, Rommelaere H (1998). Prefoldin, a chaperone that delivers unfolded proteins to cytosolic chaperonin. *Cell*.

[70] Dunn AY, Melville MW, Frydman J (2001). Review: cellular substrates of the eukaryotic chaperonin TRiC/CCT. *Journal of Structural Biology*.

[71] Keller CE, Lauring BP (2005). Possible regulation of microtubules through destabilization of tubulin. *Trends in Cell Biology*.

[72] Stirling PC, Cuéllar J, Alfaro GA (2006). PhLP3 modulates CCT-mediated actin and tubulin folding via ternary complexes with substrates. *Journal of Biological Chemistry*.

[73] Grantham J, Brackley KI, Willison KR (2006). Substantial CCT activity is required for cell cycle progression and 
cytoskeletal organization in mammalian cells. *Experimental Cell Research*.

[74] Nolasco S, Bellido J, Gonçalves J, Zabala JC, Soares H (2005). Tubulin cofactor A gene silencing in mammalian cells induces changes in microtubule cytoskeleton, 
cell cycle arrest and cell death. *FEBS Letters*.

[75] Heald R, Nogales E (2002). Microtubule dynamics. *Journal of Cell Science*.

[76] Fletcher G, Rørth P (2007). *Drosophila* stathmin is required to maintain tubulin pools. *Current Biology*.

[77] Nguyen HL, Gruber D, Bulinski JC (1999). Microtubule-associated protein 4 (MAP4) regulates assembly, protomer-polymer partitioning and synthesis of tubulin in cultured cells. *Journal of Cell Science*.

[78] Fojo T, Menefee M (2007). Mechanisms of multidrug resistance: the potential role of microtubule-stabilizing agents. *Annals of Oncology*.

[79] Jordan CT, Guzman ML, Noble M (2006). Cancer stem cells. *New England Journal of Medicine*.

[80] Bhagra A, Rao RD (2007). Chemotheraphy-induced neuropathy. *Current Oncology Reports*.

[81] Hermelink K, Untch M, Lux MP (2007). Cognitive function during neoadjuvant chemotherapy for breast cancer: 
results of a prospective, multicenter, longitudinal study. *Cancer*.

[82] Lee H, Finck BN,  Jones LA, Welch MJ, Mach RH (2006). Synthesis and evaluation of a bromine-76-labeled PPAR*γ* antagonist 2-bromo-5-nitro-*N*-phenylbenzamide. *Nuclear Medicine and Biology*.

[83] Orr GA, Verdier-Pinard P, McDaid H, Horwitz SB (2003). Mechanisms of Taxol resistance related to microtubules. *Oncogene*.

[84] Coghlin C, Carpenter B, Dundas SR, Lawrie LC, Telfer C, Murray GI (2006). Characterization and over-expression of chaperonin t-complex proteins in colorectal cancer. *Journal of Pathology*.

[85] Seiden-Long IM, Brown KR, Shih W (2006). Transcriptional targets of hepatocyte growth factor signaling and Ki-*ras* oncogene 
activation in colorectal cancer. *Oncogene*.

[86] Yokota S, Yamamoto Y, Shimizu K (2001). Increased expression of cytosolic chaperonin CCT in human hepatocellular and colonic carcinoma. *Cell Stress & Chaperones*.

[87] Xiao L, Lu X, Ruden DM (2006). Effectiveness of Hsp90 inhibitors as anti-cancer drugs. *Mini Reviews in Medicinal Chemistry*.

[88] Powers MV, Workman P (2006). Targeting of multiple signalling pathways by heat shock protein 90 molecular chaperone inhibitors. *Endocrine-Related Cancer*.

[89] Welch JS, Ricote M, Akiyama TE, Gonzalez FJ, Glass CK (2003). PPAR*γ* and PPAR*δ* negatively regulate specific subsets of lipopolysaccharide and IFN-*γ* target genes in macrophages. *Proceedings of the National Academy of Sciences of the United States of America*.

[90] Pasquier E, André N, Braguer D (2007). Targeting microtubules to inhibit angiogenesis and disrupt tumour vasculature: 
implications for cancer treatment. *Current Cancer Drug Targets*.

[91] Pasquier E, Honoré S, Braguer D (2006). Microtubule-targeting agents in angiogenesis: where do we stand?. *Drug Resistance Updates*.

[92] Talmadge JE, Donkor M, Scholar E (2007). Inflammatory cell infiltration of tumors: Jekyll or Hyde. *Cancer and Metastasis Reviews*.

[93] Allavena P, Sica A, Solinas G, Porta C, Mantovani A (2008). The inflammatory micro-environment in tumor progression: 
the role of tumor-associated macrophages. *Critical Reviews in Oncology/Hematology*.

[94] Mantovani A, Sozzani S, Locati M, Allavena P, Sica A (2002). Macrophage polarization: tumor-associated macrophages as a paradigm for polarized M2 mononuclear phagocytes. *Trends in Immunology*.

[95] Bouhlel MA, Derudas B, Rigamonti E (2007). PPAR*γ* activation primes human monocytes into alternative M2 macrophages with anti-inflammatory 
properties. *Cell Metabolism*.

[96] Hontecillas R, Bassaganya-Riera J (2007). Peroxisome proliferator-activated receptor *γ* is required for regulatory CD4^+^ T cell-mediated protection against colitis. *Journal of Immunology*.

[97] Curiel TJ (2007). Tregs and rethinking cancer immunotherapy. *Journal of Clinical Investigation*.

